# Comprehensive analysis of prognostic and immunological role of basement membrane‐related genes in soft tissue sarcoma

**DOI:** 10.1002/iid3.70037

**Published:** 2024-10-11

**Authors:** Guang‐hua Nie, Cheng‐yi Liu, Zhao Tian

**Affiliations:** ^1^ Department of Foot and Ankle Surgery, Honghui Hospital Xi'an Jiaotong University Xi'an China; ^2^ Department of Hand Surgery, Honghui Hospital Xi'an Jiaotong University Xi'an China

**Keywords:** basement membrane, prognosis, signature, soft tissue sarcoma, tumor microenvironment

## Abstract

**Background:**

Soft tissue sarcoma (STS) represents highly multifarious malignant tumors that often occur in adolescents and have a poor prognosis. The basement membrane, as an ancient cellular matrix, was recently proven to play a vital role in developing abundant tumors. The relationship between basement membrane‐related genes and STS remains unknown.

**Methods:**

Consensus clustering was employed to identify subgroups related to differentially expressed basement membrane‐related genes. Cox and least absolute shrinkage and selection operator regression analyses were utilized to construct this novel signature. Then, we established a nomogram and calibration curve, including the risk score and available clinical characteristics. Finally, we carried out functional enrichment analysis and immune microenvironment analysis to investigate enriched pathways and the tumor immune microenvironment related to the novel signature.

**Results:**

A prognostic predictive signature consisting of eight basement membrane‐related genes was established. Kaplan–Meier survival curves demonstrated that the patients in the high‐risk group had a poor prognosis. Independent analysis illustrated that this risk model could be an independent prognostic predictor. We validated the accuracy of our signature in the validation data set. In addition, gene set enrichment analysis and immune microenvironment analysis showed that patients with low‐risk scores were enriched in some pathways associated with immunity. Finally, in vitro experiments showed significantly differential expression levels of these signature genes in STS cells and PSAT1 could promote the malignant behavior of STS.

**Conclusions:**

The novel signature is a promising prognostic predictor for STS. The present study may improve the prognosis and enhance individualized treatment for STS in the future.

## 1 INTRODUCTION

Soft tissue sarcoma (STS) represents a group of highly malignant tumors, which is aggressive and heterogeneous, derive from mesenchymal tissues, and can occur in various parts of the body.^1^ Although STS accounts for approximately 1% of adult malignancies, its incidence is relatively higher among adolescents.^2^ At present, the therapeutic strategies for STS include surgery, radiotherapy, and chemotherapy.^3^ Despite notable advancements in localized treatments such as surgery and radiotherapy, a significant proportion of patients still experience tumor recurrence and metastasis.^4^ At the terminal stage of STS, the efficiency of comprehensive therapy remains limited and is associated with poor survival outcomes.^5^ With the advent of immunotherapies^6^ and their effectiveness in other tumor types such as Ewing sarcoma and osteosarcoma,^7^, ^8^ the identification of reliable biomarkers are vital for early diagnosis, chemotherapy prediction, and prognosis prediction for patients with STS. Given these limitations, early and accurate diagnosis and prognosis prediction are critical for treating and improving overall survival (OS) in STS.

Basement membrane (BM) represents an ancient kind of extracellular matrix that exists widely in various animal cells. It is established on the network of laminin and the type IV collagen and is of importance in the structure and signaling of many tissues.^9^ At present, many studies reported that BM participated in many signs of progression, such as diabetes, hypertension, and cancer.^10^ For malignant cells to metastasize, they must breach BM barriers and recruit an abundant vascular supply.^11^ Malignancy can change their integrin signaling patterns to adapt to foreign extracellular matrix or overexpress extracellular matrix‐related protein to live.^12^ Recently, Ranjay^13^ et al. identified more than 100 BM‐related genes (BMRG) that impact the immense complexity of BMs and human health. The genes discovered in their study may provide new biomarkers or pathways for STS diagnosis, prognosis prediction, and treatment. To date, no studies focused on the association between BMRG and STS.

In our study, a comprehensive bioinformatic analysis and experiments were performed to investigate the clinical relevance, prognostic performance, and immunological role of BMs in STS. As a result, this study revealed the significance of BMRGs in STS, providing a basis for prognosis prediction, identification of immunity features, chemotherapy, and immunotherapy strategies.

## 2 METHODS

### 2.1 Data sets

The Cancer Genome Atlas (TCGA) is an open data library containing genomic sequences and gene expression for abundant tumors.^14^ The Gene Expression Omnibus (GEO) is an open repository containing high throughput expression and other functional genomics data sets, freely supplied to researchers.^15^ For the following analyses, the data on STS patients' gene expression and clinical characteristics are from the two databases above. Patients with incomplete or difficult‐to‐identify clinical characteristics were excluded. A total of two data sets, TCGA data sets including 259 patients and GSE71118 from GEO including 312 patients, were obtained in this trial. Furthermore, the meta‐cohort of STS cohorts was generated using “Combat” algorithm. The clinical information was displayed in Supporting Information S2: Table S1.

### 2.2 BMRG consensus clustering

Two hundred twenty‐three corresponding BMRGs were retrieved from previous studies^13^ as candidate gene sets. Supporting Information S2: Table S2 illustrates detailed information about these genes. Consensus clustering was carried out to identify distinct subgroups based on BMRG expression. The R package “ConsensusClusterPlus” was carried out to initiate this analysis.^16^ To further examine the differences between the distinct clusters, we generated a heatmap comparing the patient characteristics. Subsequently, to determine biological processes of these genes, gene set variation analysis (GSVA) was initiated.^17^ Association between the clinical features and two distinct clusters was explored via survival analysis, heatmap, and principal component analysis (PCA).

### 2.3 Immune microenvironment analysis

ESTIMATE algorithm was carried out to evaluate the immune scores, stromal scores, and ESTIMATE scores. Subsequently, single‐sample gene set enrichment analysis was performed to investigate immune cell infiltration levels and assess their function in the STS tumor microenvironment (TME). Furthermore, immune infiltration levels of 22 human cells were evaluated through CIBERSORT algorithm.^18^

### 2.4 Identification of differentially expressed genes (DEGs)

Threshold setting as |log2FC | ≥1 and false discovery rate < 0.05 was established to identify DEGs among distinct subtypes using “limma” package.^19^ To further explore enriched signaling pathways and the potential functional mechanism, we initiated Gene Ontology (GO) and Kyoto Encyclopedia and Genomes (KEGG) enrichment analyses.^20^

### 2.5 Establishing and validating a prediction model

To screen out the DEBM cluster‐related genes that related to STS prognosis, we carried out univariate Cox regression analysis under inclusion threshold of *p* < .05. Then, the entire group (*n* = 259) was equally assigned to training cohort (*n* = 131), testing cohort (*n* = 128). After that, STS patients were assigned to accordingly gene clusters according to the result of consensus clustering from expression of above‐identified DEBM cluster‐related genes. Furthermore, we carried out the least absolute shrinkage and selection operator (LASSO) Cox regression analysis to further screen differentially expressed BM cluster‐related genes for signature construction. To calculate included genes and corresponding coefficients, respectively, for the following establishment of this predictive signature, multivariate Cox regression was initiated. The risk score calculation formula is as follows: Risk score = Σ (Expi * coefi). After that, patients were categorized into high‐ and low‐risk groups based on median risk score of training cohort, respectively. Then, to investigate distinctions in survival status among risk groups and evaluate efficiency of the predictive signature, Kaplan–Meier (K–M) survival curve, receiver operating characteristic (ROC) curves, and heatmaps of gene expression were generated in training, testing, entire, and GSE71118 cohorts, respectively.

### 2.6 Stratification analyses of prognostic risk scores

To explore the relationship between risk scores and clinical features including age, margin status, gender, metastasis, and new tumor events, chi‐square test was conducted. After that, we initiated univariate and multivariate Cox analyses to explore if the risk scores were not related to other clinical characteristics in training cohort. Furthermore, stratified analysis was employed to investigate if risk score maintained the efficiency of prediction across distinct risk groups.

### 2.7 Establishment and validation of a nomogram scoring system

We further generated scoring system consisting of clinical features and risk scores. Each variable corresponds to its score one by one, and the total score serves as the summarized score of all variables in each sample. To further assess the predictive efficiency, 1‐, 3‐, and 5‐year survival ROC curves were generated. Meanwhile, calibration curves were generated to explore whether the above results of survival predictions were consistent with the actual results.

### 2.8 GSEA

As an effective tool exploring gene functional enriched pathways in certain gene sets,^21^ GSEA was used to determine enrichment levels of biological processes, revealing biological mechanisms of the genes included. The c2.cp.kegg.v7.4.symbols.gmt in Molecular Signatures Database was selected as the reference gene collections in GSEA software. The top five enrichment pathways in two distinct risk groups were selected for illustration, respectively.

### 2.9 Mutation and drug sensitivity analysis

We generated mutation annotation format considering the recognized important role of somatic mutations in tumor cell migration and proliferation through “maftools” package.^22^ Furthermore, tumor mutation burden (TMB) score was determined. Considering chemotherapy plays a vital role in abundant tumors, we assessed semi‐inhibitory concentration (IC50) of chemotherapeutical medications selected from commonly used drugs for tumors via “pRRophetic” package.^23^

### 2.10 Cell culture

Human synovial sarcoma cell line (SW982) was sourced from the American Type Culture Collection. The human skin fibroblast cell line was purchased from Fenghui Biotechnology Co., Ltd. (Hunan, China). The human liposarcoma cell line (SW872) and synovial sarcoma cell line (SYO‐1) were acquired from Procell Life Science & Technology Co., Ltd. (Hubei, China). All cell lines were cultured in Dulbecco's modified Eagle's medium (Gibco, 11965118), with 10% fetal bovine serum (Gibco, A5670701) and 1% penicillin‐streptomycin solution (New Cell & Molecular Biotech, C100C5). Cells were maintained at 37°C in moist air containing 5% CO_2_.

### 2.11 Cell transfection

Inoculate cells onto a six‐well plate and transfect when 30%–50% of cells fusion. Mix a solution containing 250ul Opti MEM (Gibco, 51985091) and 5 μL siRNA (20 μM) with another solution containing 250 μL Opti MEM and 5 μL lipo2000 (Invitrogen, 11668019). Mix the solution with corresponding cells, shake well. Cells were cultured at 37°C, and after 6–8 h, the complete culture medium was replaced, and appropriate cultivation was carried out for subsequent experiments. The siRNA sequences are as follow: siPSAT1#1: 5′‐CCCUAAACUUGGGAGUUAU‐3′, siPSAT1#2: 5′‐ACTCAGTGTTGTTAGAGAT‐3′. The siRNA was purchased from the Hanheng Biotechnology (Shanghai, China).

### 2.12 Real‐time quantitative polymerase chain reaction (RT‐qPCR)

Under the guidance of the manufacturer's protocol, total cellular RNA was extracted using RNA Express Total RNA Kit (New Cell & Molecular Biotech, M5100). Subsequently, the Hifair® III 1st Strand cDNA Synthesis SuperMix for qPCR (Yeasen Biotechnology Co., Ltd., 11141ES60). was applied for cDNA synthesis. Finally, the gene expression was quantified by Hieff qPCR SYBR Green Master Mix (High Rox Plus) (YEASEN Biotech Co., Ltd., 11203ES) and calculated with the $2^{-∆∆C_{t}}$ method. GAPDH acted as an internal reference. The primer sequences used are presented in Supporting Information S2: Table S3.

### 2.13 Cell count kit‐8 (CCK‐8) assay

CCK‐8 reagent (New Cell & Molecular Biotech, China, C6005) was drawn into the 96 well plate, which was previously filled with SW982 and SYO‐1 cells (1000 cells/well) at intervals of 24, 48, 72, 96, and 120 h. Place the plate in cell culture incubator. Use a microplate reader (Biotek Microplate spectrophotometer, Epoch 2) to measure the optical density of 450 nm for each well.

### 2.14 Colony formation assay

Two cell lines (SW982 and SYO‐1) are distributed on six well plates. Culture for 7–10 days, then wash with phosphate buffered saline (PBS), cells fixation, stain, and finally, counting was performed by ImageJ.

### 2.15 Wound healing assay

SW982 and SYO‐1 cells were inoculated onto a six‐well plate until fusion reached 90%. Gently scrape with the tip of a 200 μL pipette to form a linear incision. Wash the sample with PBS, eliminating loose cells. Photos were taken at two time points, especially 0 and 24 h after the experimental procedure. ImageJ was used to measure the wound healing rate.

### 2.16 Statistical analysis

R (V4.0.2) was employed for analysis, and the results of in vitro experiments were analyzed through GraphPad Prism (version 9) and ImageJ. Wilcoxon test was utilized to compare differences between groups, while the Kruskal–Wallis and one‐way analysis of variance tests were employed to analyze differences among over three groups. The Spearman analysis was performed to determine correlations between variables. For each analysis, a *p* < .05 was considered statistically significant.

## 3 RESULTS

### 3.1 Alteration of BMRGs in STS

**Figure 1:**
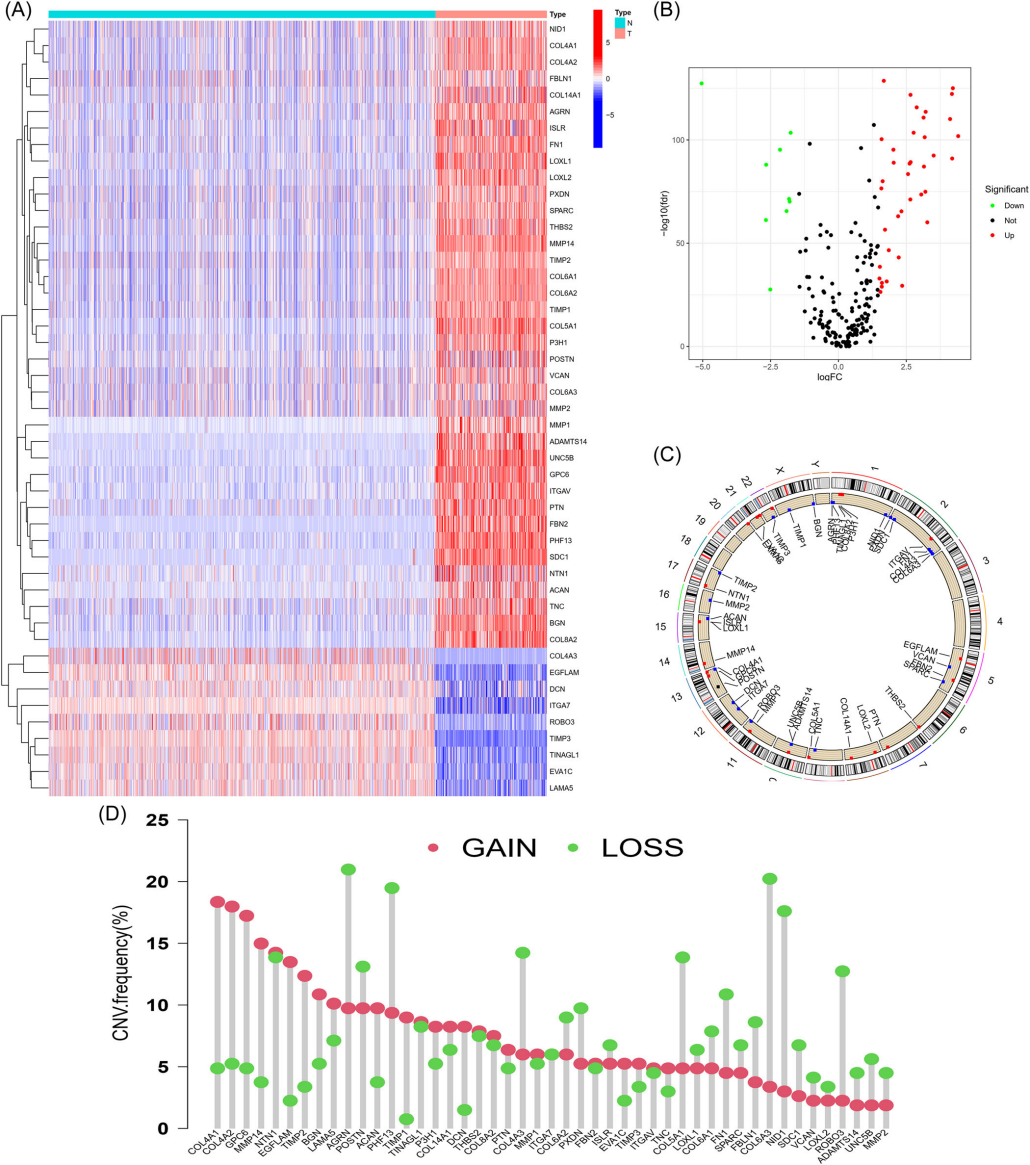
Genetic and transcriptional alterations of BMRGs in STS. (A) Expression distributions of BMRGs between normal and STS tissues. (B) Volcano map of the expression of genes. (C) Locations of CNV alterations in BMRGs on 23 chromosomes. (D) Frequencies of CNV gain, loss, and non‐CNV among BMRGs. BMRGs, basement membrane‐related genes; CNV, copy number variant; STS, soft tissue sarcoma; TCGA, The Cancer Genome Atlas.

To explore expression of BMRGs in STS, we initiated differential analysis using “limma” package. Forty‐seven DEBMRGs were screened out. The heatmap and volcano plot of the 47 DEBMRGs was shown in Figure 1A,B. Given their abnormal expression in STS, we then investigated the frequency of somatic mutations in these 47 differentially expressed BMRGs. As shown in the waterfall map, 38 of 238 (15.97%) patients had mutations in these genes (Supporting Information S1: Figure S1), among which the top five mutated genes with their mutation frequencies were ACAN (3%), COL4A1 (2%), COL6A3 (2%), FBN2 (2%), and TNC (2%). After that, we further explored the copy number variation (CNV) of alteration. The chromosome locations of CNV alterations of BMRGs were displayed in Figure 1C. All 47 differentially expressed BMRGs had CNV alterations (Figure 1D). COL4A1, COL4A2, and GPC6 had prevalent CNV amplification, while AGRN, PHF13, COL6A3, and NID1 had CNV decreases.

### 3.2 BMRG clustering

**Figure 2:**
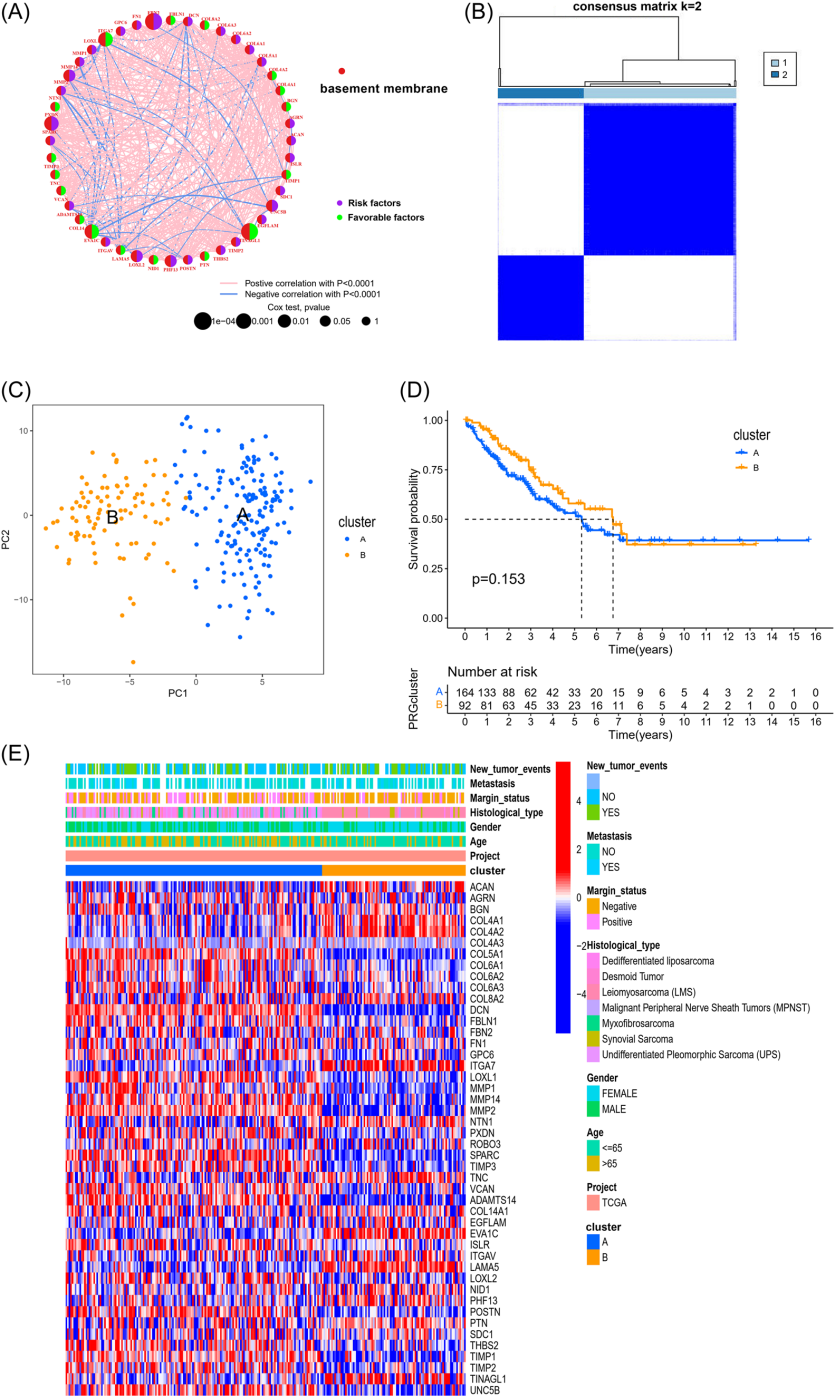
BMRG subtypes and clinicopathological and biological characteristics of two distinct subtypes of samples divided by consistent clustering. (A) Interactions among BMRGs in STS. The line connecting the BMRGs represents their interaction, with the line thickness indicating the strength of the association between BMRGs. Green and pink represent negative and pink positive correlations, respectively. (B) Consensus matrix heatmap defining two clusters (k = 2) and their correlation area. (C) PCA analysis showing a remarkable difference in transcriptomes between the two subtypes. (D) Kaplan–Meier curves for OS of two distinct molecular subtypes (log‐rank tests, *p *= .153). (E) Differences in clinicopathologic characteristics and expression levels of BMRGs between the two distinct subtypes. BMRGs, basement membrane‐related genes; OS, overall survival; PCA, principal components analysis; STS, soft tissue sarcoma.

We investigated the effects of these 47 DEBMRGs on the prognosis of STS. Univariate Cox regression and K–M analysis were carried out, revealing value of 47 BMRGs for prognosis (Supporting Information S2: Table S4). Next, the network illustrated the landscape of BMRG interactions, regulator connections, and their prognostic efficiency in STS patients (Figure 2A and Supporting Information S2: Table S5). To investigate expression characteristics of BMRGs in STS, a consensus clustering method was carried out to categorize the STS patients based on BMRGs expression. As a result, from 1 to 9, k = 2 exhibited to be the best selection, that is, categorizing the STS patients into two clusters (Figure 2B). After that, PCA was employed to reveal difference between distinct groups in the transcription of the BMRGs, and the result showed a significant difference as predicted (Figure 2C). Meanwhile, we performed K–M analysis, demonstrating no marked difference in survival probability between two subtypes (Figure 2D). Also, a heatmap revealing differences between two clusters in gene expression and clinical features was generated (Figure 2E).

### 3.3 Characteristics of the TME in STS

**Figure 3:**
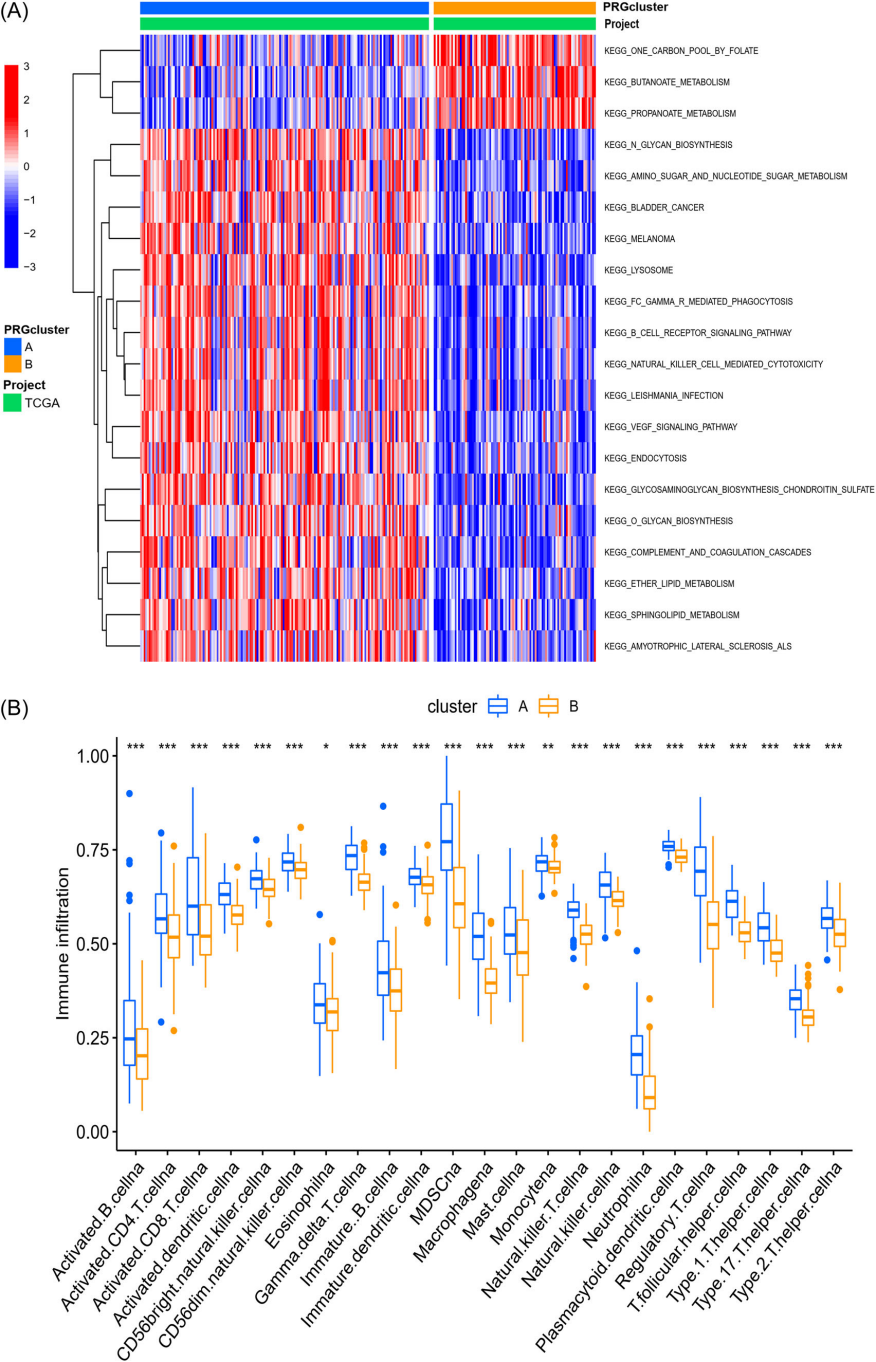
Correlations of tumor immune cell microenvironments and two STS subtypes. (A) GSVA of biological pathways between two distinct subtypes, in which red and blue represent activated and inhibited pathways, respectively. (B) The abundance of 22 infiltrating immune cell types in the two STS subtypes. GSVA, gene set variation analysis; STS, soft tissue sarcoma.

To investigate enriched pathways and potential biological processes between the two distinct BM‐related clusters, we initiated GSVA enrichment analysis, revealing cluster A was markedly enriched in some oncogenic‐activated and immune‐activated pathways, such as bladder cancer, melanoma, NK cell‐mediated cytotoxicity, B cell receptor signaling pathway. In comparison, cluster B was enriched significantly in some metabolism pathways, such as one carbon pool by folate, butanoate metabolism, and propanoate metabolism (Figure 3A, Supporting Information S2: Table S6). Also, we explored mechanism of immune infiltration in STS in the two groups, and we explored relationships between the groups and immune cells using CIBERSORT (Supporting Information S2: Table S7). Meanwhile, a box plot comparing infiltration levels of the human immune cells was generated (Figure 3B). Results displayed a marked difference in infiltration between two clusters. Higher filtration levels were detected in cluster A for all the human immune cells.

### 3.4 Identification of gene clusters

**Figure 4:**
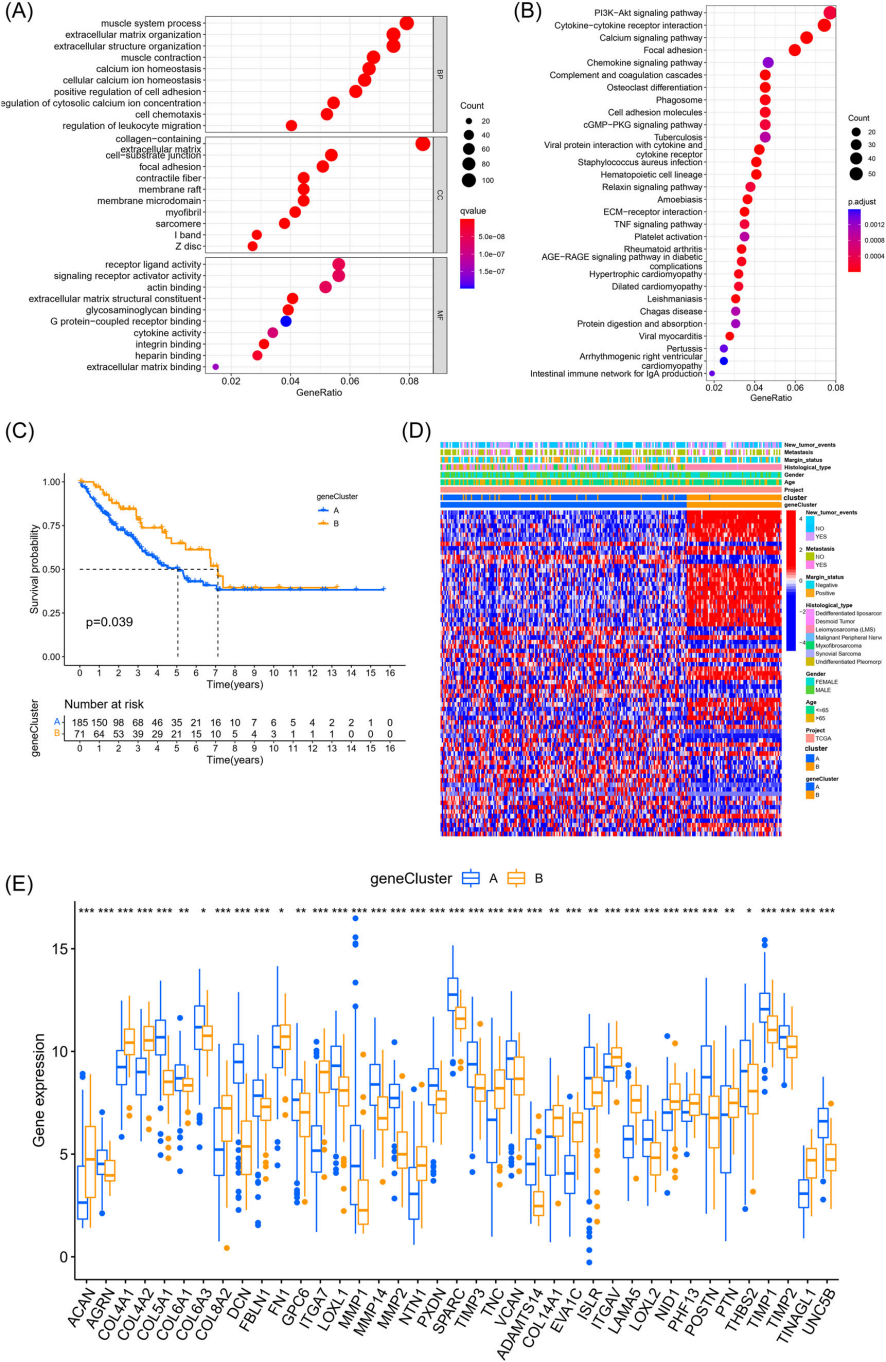
Identification of gene subtypes based on DEGs. (A) GO enrichment analysis of differentially expressed basement membrane cluster‐related genes. (B) KEGG enrichment analysis of differentially expressed basement membrane cluster‐related genes. (C). Kaplan–Meier curves for OS of the two gene subtypes (log‐rank tests, *p *< .05). (D) Relationships between clinicopathologic characteristics and the two gene subtypes. (E) Differences in the expression of BMRGs among the two gene subtypes. BMRGs, basement membrane‐related genes; DEG, differentially expressed gene; GO, gene ontology; KEGG, Kyoto Encyclopedia and Genomes; OS, overall survival.

We screened out 1472 BM cluster‐related DEGs by differential analysis, based on previous studies. At the same time, functional enrichment analyses were carried out based on above BM cluster‐related DEGs. The GO enrichment analysis was shown in Figure 4A and Supporting Information S2: Table S8, these BM cluster‐related DEGs were enriched in muscular process and extracellular matrix organization. The KEGG enrichment results exhibited that these genes were enriched in PI3K‐Akt signaling pathway, cytokine–cytokine receptor interaction, and calcium signaling pathway (Figure 4B, Supporting Information S2: Table S8). Subsequently, univariate Cox regression was initiated according to the 1472 BM cluster‐related DEGs to evaluate the prognostic efficiency. As a result, 73 genes associated with OS of STS were screened out (Supporting Information S2: Table S9). Furthermore, to validate the mechanism, we utilized these 73 genes to divide patients with STS into two gene clusters (Supporting Information S1: Figure S2). Also, K–M curves were generated, indicating that patients in cluster B have a better OS (*p *< .05, Figure 4C). Heatmap exhibited differences in clinical features between subtypes (Figure 4D). Furthermore, the box plot indicated marked differences in gene expression levels between two gene clusters (Figure 4E).

### 3.5 Establishment and validation of a prognostic signature

**Figure 5:**
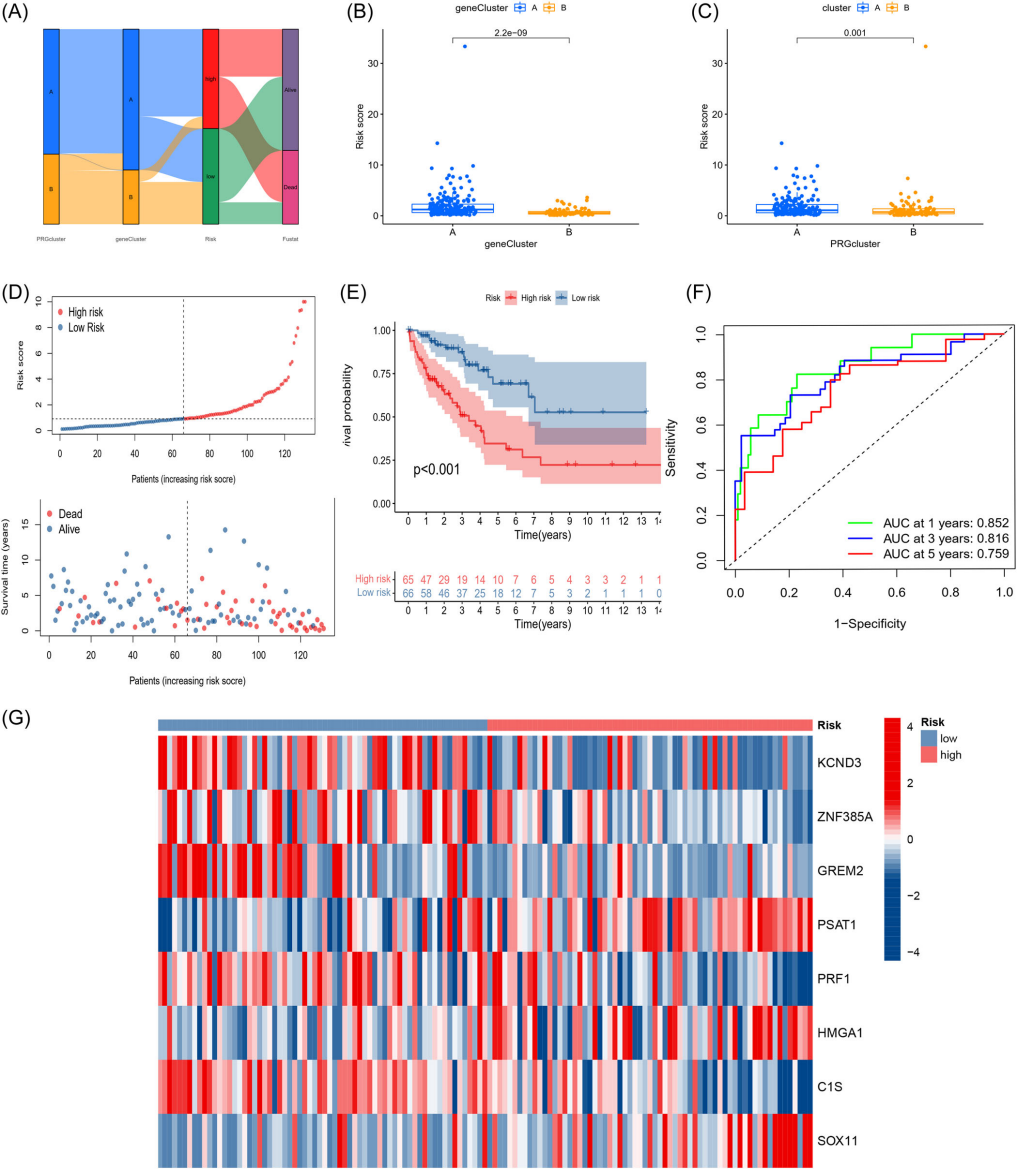
Construction of the risk score in the training set. (A) Alluvial diagram of subtype distributions in groups with different risk scores and survival outcomes. (B) Differences in risk score between gene subtypes. (C) Differences in risk score between the two subtypes. (D) Ranked dot and scatter plots showing the risk score distribution and patient survival status. (E) Kaplan–Meier analysis of the OS between the two groups. (F) ROC curves to predict the sensitivity and specificity of 1‐, 3‐, and 5‐survival according to the risk score. (G) Expression level of 8 signature‐related genes. OS, overall survival; ROC, receiver operating characteristic.

We initiated LASSO analysis based on 1472 BM cluster‐related DEGs and remained 14 cluster‐related DEGs on the basis of minimum partial likelihood deviance. To find optimum prognostic signature, multivariate Cox regression on above 14 OS‐related DEGs was performed on the basis of the Akaike information criterion value, and ultimately, eight genes remained (Supporting Information S1: Figure S3). Therefore, a signature containing eight genes, KCND3, ZNF385A, GREM2, PSAT1, PRF1, HMGA1, C1S, SOX11, was established, and formula for risk score was as follows: Risk score = (KCND3 × −0.1773) + (ZNF385A × − 0.2505) + (GREM2 × −0.2782) + (PSAT1 × 0.1321) + (PRF1 × −0.2365) + (HMGA1 × 0.1576) + (C1S × −0.1498) + (SOX11 × 0.0733). The distribution of patients in the two BMRG clustering, two gene clustering, and two risk groups was shown in Figure 5A. Then, we investigated the differences between the two clusters and two gene clusters in risk scores, respectively, and the results indicated markedly higher risk scores in cluster A (Figure 5B). Similarly, gene cluster A had higher risk scores (Figure 5C). We further investigated distribution of risk scores and survival status (Figure 5D), indicating that as the risk score increases, survival time decreases and the death rate increases. The K–M curve revealed that STS patients had a markedly better OS in low‐risk group (*p *< .05, Figure 5E). Furthermore, the areas under curves of the ROC curve were 0.852, 0.816, and 0.759 at 1‐, 3‐, and 5‐ year (Figure 5F), indicating that the novel signature was successfully constructed, and it has a practical value in predicting STS patients' survival. The eight genes expressions in the cohort were shown in Figure 5G. To further validate the value of the predictive potency of the signature, a validation analysis of the test cohort and the entire cohort was performed as the internal validation and the GEO cohort (GSE71118) was used as the external validation. Similar to the training group, the other three groups used for validation were also categorized into high‐ and low‐risk groups, and then risk plots, K–M survival curves, and ROC are successfully generated. Accordingly, a validation analysis of the three groups indicated that the novel signature had effective capacity in the prognostic prediction in STS patients (Supporting Information S1: Figure S4).

### 3.6 Prediction value of the signature

Stratified analysis were employed to investigate predictive ability of the risk scores in abundant clinical characteristic groups, including age (≤60 and >60 years), gender (female and male), margin status (negative and positive), metastasis (no and yes), and new tumor event (no and yes). Patients with high risk scores have lower OS in age (*p *< .001 for >60 years and *p *= .006 for ≤60 years), gender (*p *< .001 for female and *p *= .002 for male), margin status (*p *= .003 for negative and *p *< .001 for positive), metastasis (*p *< .001 for no and *p *= .010 for yes), and new tumor event (*p *< .001 for no and *p*= .003 for yes) (Supporting Information S1: Figure S5a–j). The HR of the BMRG risk level was 1.174 (95% confidence interval [CI]: 1.118–1.233) and 1.147 (95% CI:1.084–1.213) in univariate and multivariate Cox regression, respectively (Supporting Information S1: Figure S5k,l). Both univariate and multivariate Cox regression exhibited that risk scores were markedly related to STS prognosis.

### 3.7 Enrichment analysis

The GSEA exhibited the differences in biological processes between risk groups. STS patients in high‐risk group were enriched in axon guidance, hedgehog signaling pathway, basal cell carcinoma, ribosome, and transfoming growth factor‐β (TGF‐β) signaling pathway. Those in low‐risk group were markedly enriched in cytokine‐cytokine receptor interaction, chemokine signaling pathway, graft versus host disease, hematopoietic cell lineage, and immunodeficiency (Supporting Information S1: Figure S6).

### 3.8 Evaluation of TME and checkpoints between two risk groups

**Figure 6:**
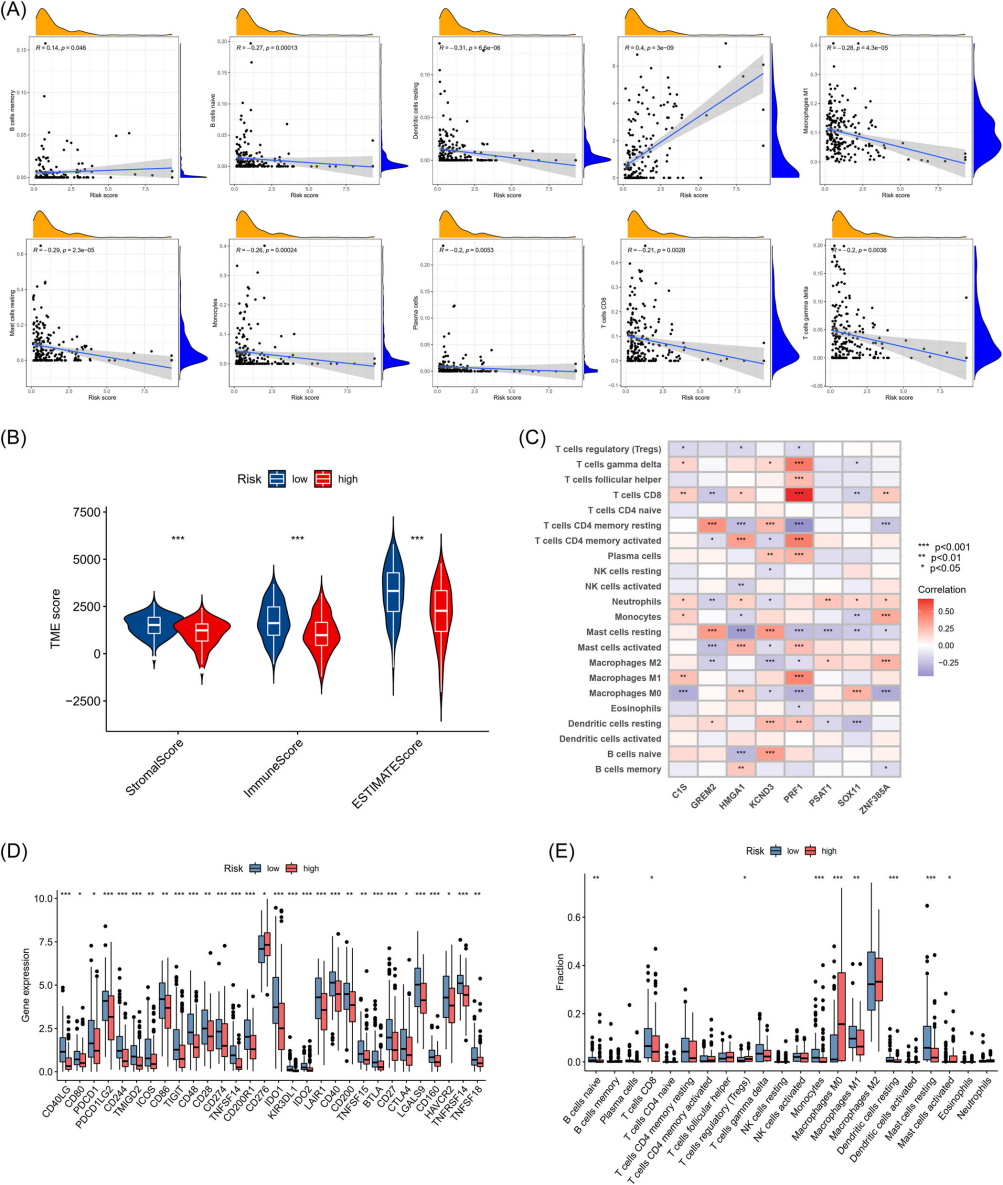
Evaluation of the TME and checkpoints between the two groups. (A) Correlations between risk score and immune cell types. (B) Correlations between risk score and immune, stromal, and ESTIMATE scores. (C) Correlations between the abundance of immune cells and eight genes in the proposed model. (D) Expression of immune checkpoints in the high and low‐risk groups. (E) The violin plot showed the different proportions of tumor‐infiltrating cells between the high‐risk and low‐risk groups. TME, tumor microenvironment.

CIBERSORT algorithm was performed to investigate the relationship between risk score and immune cells, revealing that risk scores were positively associated with M0 macrophages and memory B cells while negatively associated with naive B cells, resting dendritic cells, resting mast cells, monocytes, plasma cells, and CD8 + T cells (Figure 6A). To investigate the TME scores, we performed violin plots. As shown in the plots, the stromal score, immune score, and ESTIMATE score were all the better in the low‐risk group (Figure 6B). We further investigated relationships between the signature‐related BMRGs and immune cells. Results exhibited in Figure 6C revealed that abundant immune cells had significant associations with the genes. In addition, considering the importance of checkpoints, we explored immune checkpoint expressions between two risk groups, finding a marked difference in immune checkpoint expressions (Figure 6D). The association between risk scores and immune cell infiltration was also explored. Boxplots demonstrated that the infiltration levels of abundant immune cells were markedly higher in the low‐risk group (Figure 6E).

### 3.9 Relationship of risk score

**Figure 7:**
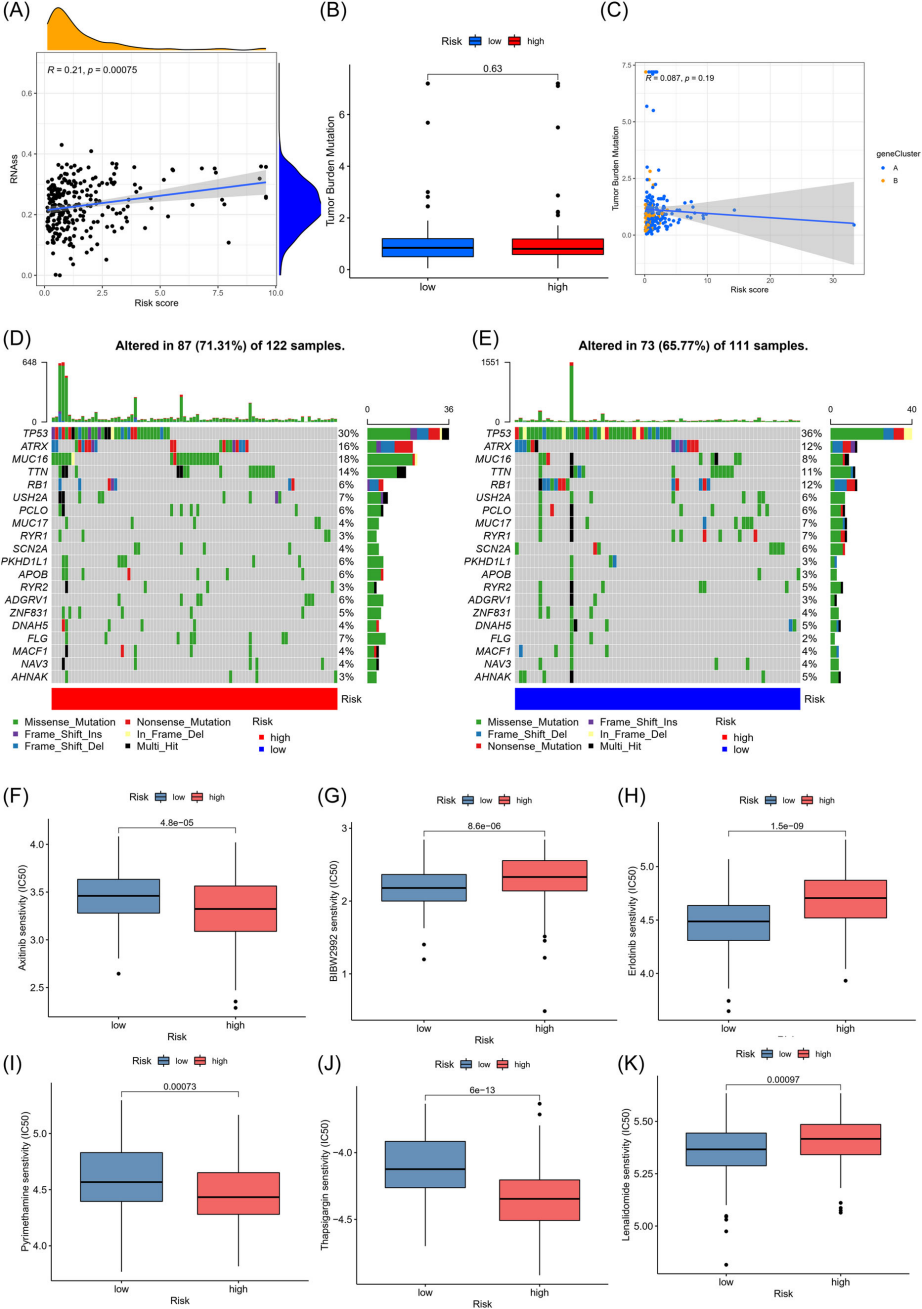
Comprehensive analysis of the risk score in STS. (A) Relationships between risk score and CSC index. (B) TMB in different risk score groups. (C) Spearman correlation analysis of the risk score and TMB. (D and E) The waterfall plot of somatic mutation features established with high and low risk scores. (F–K) Relationships between risk score and chemotherapeutic sensitivity. CSC, cancer stem cell; STS, soft tissue sarcoma; TMB, tumor mutation burden.

We assessed the potential relationship between risk scores and cancer stem cells (CSC). Risk scores were positively associated with CSC (R = 0.21, *p *< .001) (Figure 7A). We also performed the mutation analysis, showing that two risk groups have no significant difference in TMB (Figure 7B). Then Spearman correlation analysis was carried out, indicating a positive association between risk scores and the TMB (Figure 7C). After that, we found that in the high‐risk group, the most mutated genes were TP53, MUC16, ATRX, and TTN, while in low‐risk group, TP53, ATRX, RB1, and TTN were the most ones (Figure 7D,E). Mutation frequencies of TP53 and RB1 in low‐risk group were markedly higher, while those of ATRX, TTN, and MUC16 were markedly higher ones in high‐risk group. We further screened out some chemotherapy medications that were used in STS patients to evaluate the sensitivities of STS patients in the two risk groups to the commonly used medications. As a result, STS patients in low‐risk group had high IC50 values for axitinib, pyrimethamine, and thapsigargin, while those in high‐risk group were lenalidomide, bibw2992, and erlotinib (Figure 7F–K).

### 3.10 Development of a predictive nomogram

**Figure 8:**
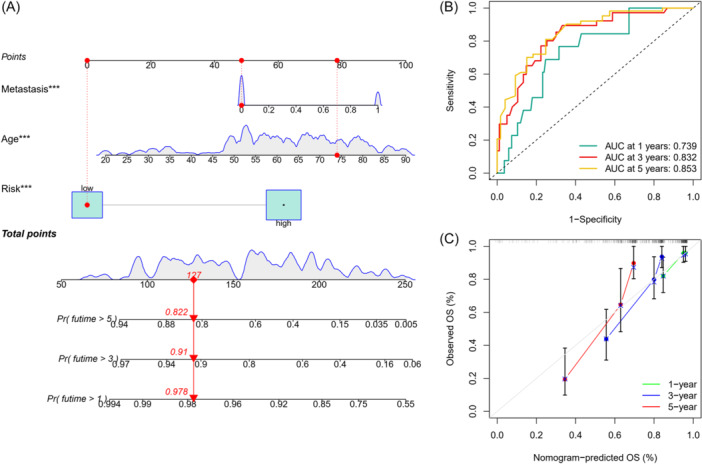
Construction and validation of a nomogram. (A) Nomogram for predicting the 1‐, 3‐, and 5‐ year OS of STS patients in the training set. (B) ROC curves for the 1‐, 3‐, and 5‐year predicted survival nomogram. (C) Calibration curve for the 1‐, 3‐, and 5‐year predicted survival nomogram. OS, overall survival; ROC, receiver operating characteristic; STS, soft tissue sarcoma.

A predictive BM‐related prognostic nomogram, including risk scores and clinical characteristics (age and metastasis), was constructed to stably and accurately predict 1‐, 3‐, and 5‐year survival (Figure 8A). Moreover, ROC of the nomogram was 0.739, 0.832, and 0.853 in 1‐, 3‐ and 5‐year, respectively. These findings validated the robustness of the prognostic signature (Figure 8B). Additionally, calibration curves were generated, and the predicted lines and actual lines overlapped well, revealing the OS predicted by the nomogram at 1‐, 3‐, and 5‐ years aligned with the observed STS patients' OS (Figure 8C).

### 3.11 Verification of the signature mRNA expression

**Figure 9:**
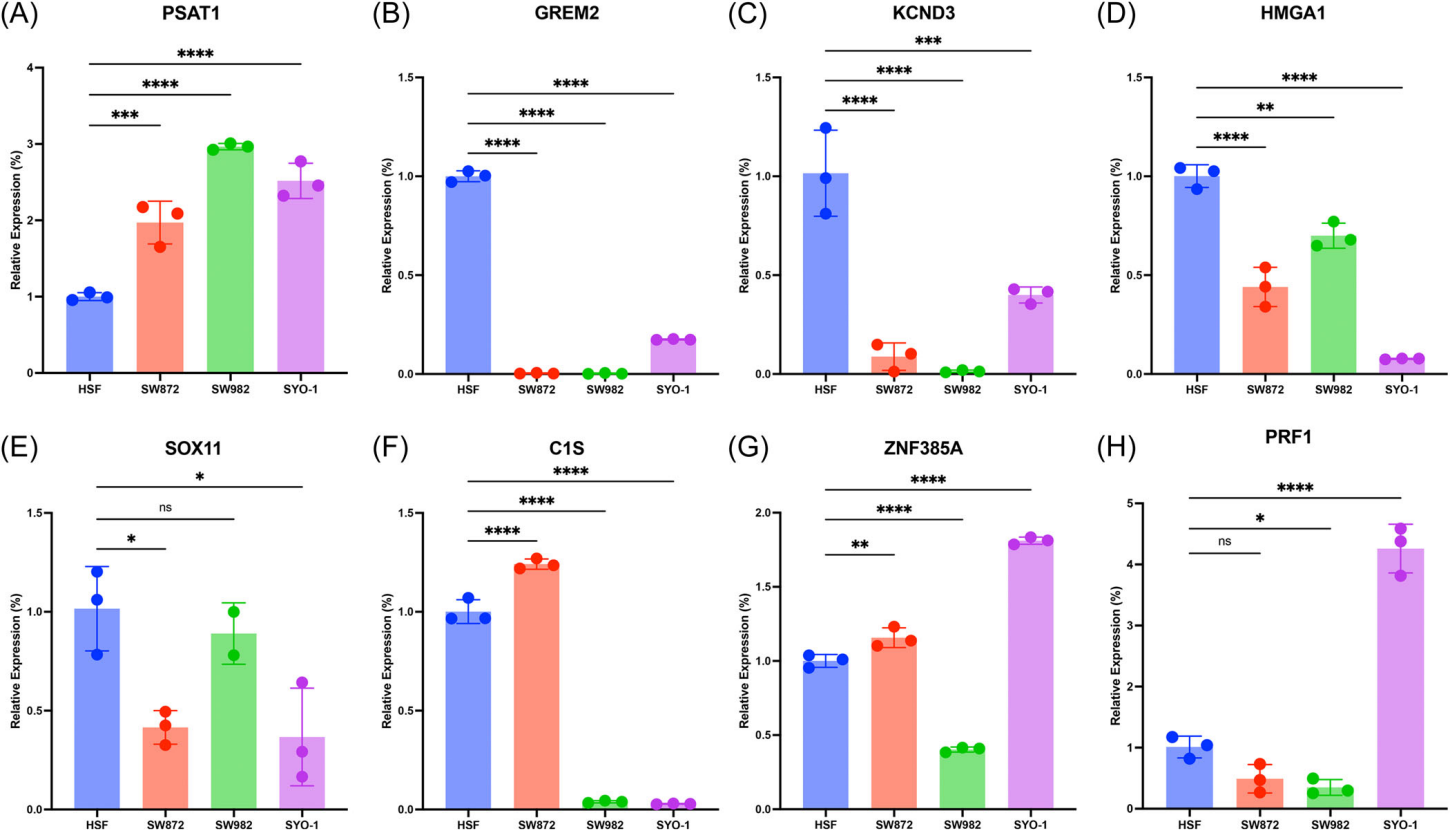
Evaluation of the expression of these eight signature BMRGs in STS cell lines. (A) PSAT1. (B) GREM2. (C) KCND3. (D) HMGA1. (E) SOX11. (F) C1S. (G) ZNF385A. (H) PRF1. *p < .05, **p < .01, ***p < .001, ****p < .0001. BMRG, basement membrane‐related genes; STS, soft tissue sarcoma.

Ultimately, we carried out RT‐qPCR to verify the eight genes expressions in STS cell lines. PSAT1 was elevated in SW982, SW872, and SYO‐1, especially in SW982 and SYO‐1 (Figure 9A). Contrary, GREM2, KCND3, HMGA1, and SOX11 were diminished (Figure 9B–E). And we found that C1S was overexpressed in SW872 while decreasing in SW982 and SYO‐1 (Figure 9F), and ZNF385A was elevated in SW872 and SYO‐1 cells but decreased in SW982 cells (Figure 9G). For PRF1, it exhibited an increasing trend in SYO‐1 (Figure 9H).

### 3.12 PSAT1 can promote the malignant progression of STS cell lines

**Figure 10:**
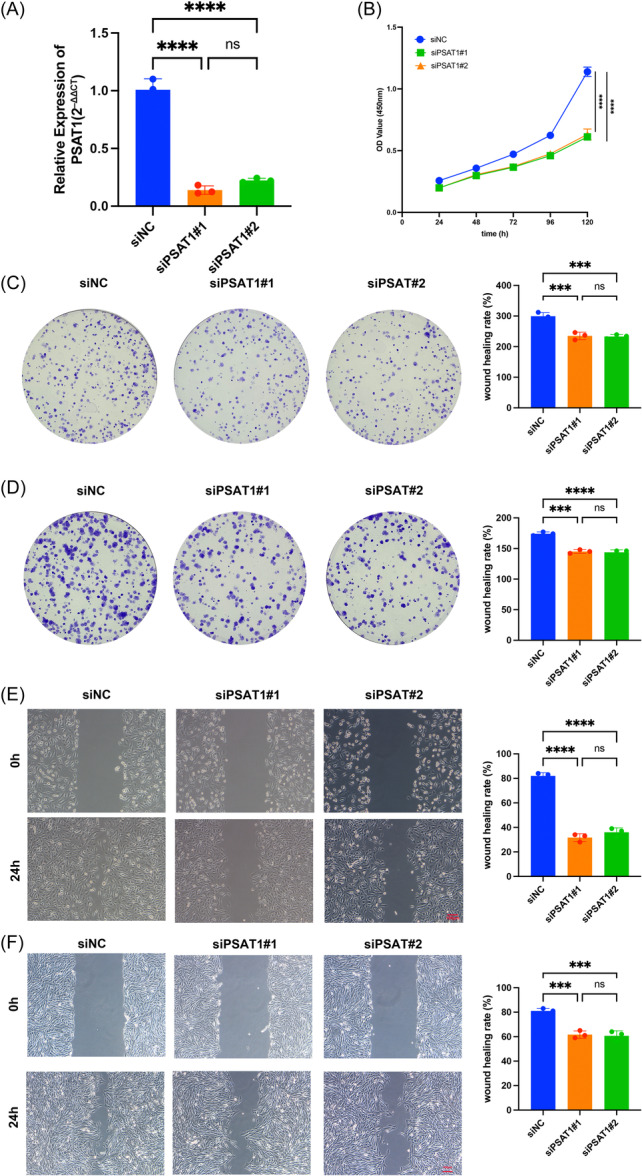
In vitro validation. (A) PCR validation of PSAT1. (B) CCK‐8 assay. (C) Colony formation assay in SYO‐1. (D) Colony formation assay in SW982. (E) Wound healing assay in SW982. (F) Wound healing assay in SYO‐1. CCK‐8, cell count kit‐8.

We selected PSAT1 for additional functional studies, as it exhibited abnormally high expression in all three STS cell lines. Initially, we interfered with PSAT1 expression by transfecting siRNA and verified the knockdown efficiency by PCR. The results exhibited that both siPSAT1#1 and siPSAT2#2 significantly downregulated PSAT1 expression in STS cells. (Figure 10A). Interfering with PSAT1 expression significantly reduces the proliferation rate of STS cells (Figure 10B). Similarly, we performed a colony formation assay on SW982 and SYO‐1 cell lines. The results showed that after downregulation of PAST1, both the number and size of colonies formed by STS cell lines significantly decreased (Figure 10C,D). Additionally, for both cell lines, we conducted wound healing assay and normal cell group had a markedly higher wound healing rate (Figure 10E,F). In summary, the above in vitro experimental results showed PSAT1 promoted the proliferation and migration ability of STS cells, further confirming the effectiveness of our analysis.

## 4 DISCUSSION

STS includes more than 80 histologic subtypes.^1^ Due to the abundance of subtypes, invasive tumor biology, and the absence of reliable biomarkers for diagnosis and treatment, STS still portends a poor prognosis.^24^ Thus, early diagnosis and treatment are critical factors in promoting STS clinical survival prognosis.^25^ At present, the BM, as an ancient cellular matrix, has been found to have certain effects on abundant biological processes. In a previous study, Jayadev R. et al. proved that BMRG provide promising insight into the prognosis prediction of tumors.^13^ However, studies focusing on the relationship between the BMRG and STS were still rare. Thus, we explored the role of BMRGs in STS and tried to construct a novel predictive signature for STS prognosis. As a result, BMRG expression was closely associated with the prognosis and TME of STS. This study is the first to reveal the relationship between BMRGs and STS.

We first investigated the expression features and genetic variation landscape of BMRGs in STS patients. From 223 BMRGs, we screened out BMRGs were differentially expressed between STS and normal tissues. Among DEBMRGs, 38 of 238 patients with STS encountered mutations with a frequency varying from 1% to 3%, among which ACAN had the highest mutation incidence in this 47 BMRGs. In previous study, ACAN was selected as a candidate biomarker for targeted therapy against gastric cancer.^26^ Subsequently, we screened out two molecular subtypes by consensus clustering based on all BMRGs expression. Patients in Cluster B had superior clinicopathological characteristics and better OS, but the difference is not significant. The GSVA analysis illustrated that cluster A was enriched in some oncogenic‐activated and immune‐activated pathways, while cluster B in some metabolism pathways. The ssGESA results illustrated a marked difference in immune cell infiltration between the two clusters. Above results demonstrated that the two subtypes had different clinical characteristics and TME. Furthermore, GO enrichment was performed, and the results demonstrated that DEBM cluster‐related genes were enriched in various important biological processes, such as the muscular process, extracellular matrix organization, extracellular structure organization, and muscle contraction. Some studies have shown that the second‐line drug, trabectedin, can treat STS not only by remodeling ECM components and cytoskeleton,^27^ but also by regulating the differentiation of monocytes and macrophages.^28^ At the same time, the current clinical research is exploring the potential clinical value of trabectedin in the treatment of STS. These results further confirmed the relationship between extracellular matrix and STS.

In addition, we established and validated the novel prognostic signature consisting of eight hub genes (KCND3, ZNF385A, GREM2, PSAT1, PRF1, HMGA1, C1S, SOX11) and categorized the patients into two risk groups. The novel signature showed a high predictive value for STS and might play a role as an independent prognostic indicator. Furthermore, ROC curve and K–M curve verified the excellent predictive ability of the BM‐related signature. We further validated the predictive potency in both external and internal cohort. A nomogram including risk score, age, and metastasis was generated. The calibration curves and ROC curve confirmed that the nomogram could effectively predict the prognosis for each STS patient.

GSEA revealed that STS patients in the high‐risk group were enriched in some tumor‐related pathways. For instance, Chen et al. found that through the TGF‐β signaling pathway, GDF15 can promote osteosarcoma migration and invasion.^29^ The Hedgehog signaling pathway plays a vital role in abundant tumors.^30^ In contrast, patients in low‐risk group were enriched in some immune‐related pathways. CXCR4 can regulate the evolution and progression of tumor cells through activating the chemokine signaling pathway.^31^ Sun et al. proved that cytokine‐cytokine receptor interaction was associated with the prognosis of bladder cancer.^32^ These results seem to remind us that tumor immunity can be associated with STS patients' prognosis. Further exploration of the relationship between tumor immunity and STS is needed.

TME is closely related to tumorigenesis, progression, and drug resistance. In this study, the different risk groups showed significantly different TME infiltration features, indicating that for low‐risk STS patients, there were more stromal cells, and immune cells, and a better TME. The results were consistent with a previous study. The infiltration abundance of many immune cells was higher in the low‐risk group, especially B cells naive, monocytes, macrophages, dendritic cells resting, and mast cells resting. Previous studies demonstrated that immune cells can play a variety of roles in tumor. For instance, macrophages affect tumorigenesis by enhancing immune cells releasing cytokine and promoting the antitumor response.^33^, ^34^ Schroeder et al. demonstrated that the activation of macrophages M2 was negatively related to OS,^35^ which is accordant with our study. Additionally, previous studies revealed that macrophage M0 was negatively associated with CD8 + T cells^36^, ^37^ and a correlation between intratumoral macrophages with lymphocyte‐to‐monocyte ratio and performance status/eastern cooperative oncology group scores.^38^ Interestingly, researchers found that high neutrophil‐to‐lymphocyte ratio, platelet‐to‐lymphocyte ratio and systemic inflammatory index were significantly associated with worse progression‐free‐survival, while low lymphocyte‐to‐monocyte ratio was significantly associated with worse OS.^39^, ^40^ The previous study and our results may explain the reason for the different prognoses of the different risk groups. The expression of immune checkpoint molecules can help tumor immune escape and can play as an efficient predictor of immunotherapy response.^41^ A recent meta‐analysis demonstrated that immune checkpoint therapy might be a promising strategy for STS treatment.^42^ In our study, the expression levels are markedly different and the expression levels in low‐risk group are higher.

We further investigated the distribution of somatic mutations and found that TP53 and ATRX were the most mutated genes in the high‐risk group. Studies have found that compared to nonmutated tumors, the 5‐year survival of STS with TP53 location missense mutations markedly reduced, indicating alteration in TP53 as effective prognostic indicator for STS.^43^ In addition, previous studies showed that ATRX is of importance in normal development. The alteration of ATRX mostly leads to the loss of ATRX. It was associated with the low differentiation of leiomyosarcoma and immune response regulation of poorly differentiated pleomorphic sarcoma, possibly through nonrecruitment of mast cells.^44^

To further explore the effect of this novel signature in the STS treatment, we explored the association between risk scores and drug sensitivity. Based on IC50 values, axitinib, thapsigargin, and pyrimethamine displayed better response in the high‐risk score group, while in the low‐risk group, erlotinib, lenalidomide, and biibw2992 demonstrated better response. These antitumor drugs have all been proven to be effective for certain cancers. For instance, a previous trial proved that axitinib plus pembrolizumab has preliminary activity in patients with advanced lung sarcomas.^45^ Additionally, pyrimethamine can inhibit cell growth by inducing cell senescence in colorectal cancer.^46^ Lenalidomide was used in combination with rituximab in patients with relapsed/refractory follicular lymphoma.^47^ These drugs can provide new insight into the treatment for STS.

Ultimately, we verified the eight BMRGs expressions in the novel signature, finding these signature genes had abnormal expression in STS, which indirectly confirmed the reliability of our analysis. At the same time, many previous studies have confirmed their role in cancer. Wang et al. established a signature containing GREM2 for clear renal cell carcinoma.^48^ We further explored the in vitro experiments to explore the impact of PSAT1 on STS. The CCK‐8 assay, and colony formation assay indicated that knocking down PSAT1 can inhibit STS cells proliferation. In addition, wound healing assays showed that knocking down PSAT1 inhibited the migration of STS cells. These experiments validated the impact of PSAT1 expression on the malignant behavior of STS. Abundant evidence indicated that PSAT1 could promote metastasis of lung adenocarcinoma,^49^ enhance cell proliferation in breast cancer,^50^ contribute to cell growth and cisplatin resistance in cervical cancer.^51^

Despite these strong findings, limitations still exist. First, due to the limited availability of clinical information in public databases and the rarity of STS, we were unable to perform such detailed stratification, which will be a focus of our future research. Second, although we verified our findings in internal and external cohorts and through RT‐qPCR, the expression levels from RT‐qPCR in different STS cell lines were not entirely consistent. Since STS refers to a group of highly heterogeneous tumors, validation tests in different cell lines may show potential inconsistencies. Therefore, we need to collect different clinical paired STS samples to further validate the transcriptional or protein levels of these novel signature genes in each type of STS in the future. For instance, experiments such as Western blot or immunohistochemistry are important methods to further validate these results. Meanwhile, the role of these genes in STS and the related mechanisms need to be the focus of future studies.

## 5 CONCLUSIONS

This study revealed BMRG's extensive regulatory mechanism affecting TME, clinicopathological characteristics, and prognosis in STS. Moreover, an eight‐hub gene signature can accurately predict the prognosis of STS patients. These results highlight vital clinical significance of BMRGs and might provide new strategies for predicting clinical prognosis and therapy of STS.

## AUTHOR CONTRIBUTIONS

**Guang‐hua Nie**: Data analysis; write—original. **Cheng‐yi Liu**: Data analysis; write–reviewing. **Zhao Tian**: Write–reviewing.

## ETHICS STATEMENT

All authors have provided their consent for the publication of this article.

## Supporting information

Supporting information.

Supporting information.

Supporting information.

## Data Availability

The data sets used in the current study are openly available in TCGA (https://cancergenome.nih.gov/) and GEO (https://www.ncbi.nlm.nih.gov/geo/). All data and materials are available from the corresponding authors upon request.
