# Supplementary figures and images for: Comprehensive analysis of prognostic and immunological role of basement membrane‐related genes in soft tissue sarcoma
Source: Immun Inflamm Dis. 2024 Oct 11;12(10):e70037. doi: 10.1002/iid3.70037 (PMC11467964; doi:10.1002/iid3.70037)

Altered in 38 (15.97%) of 238 samples

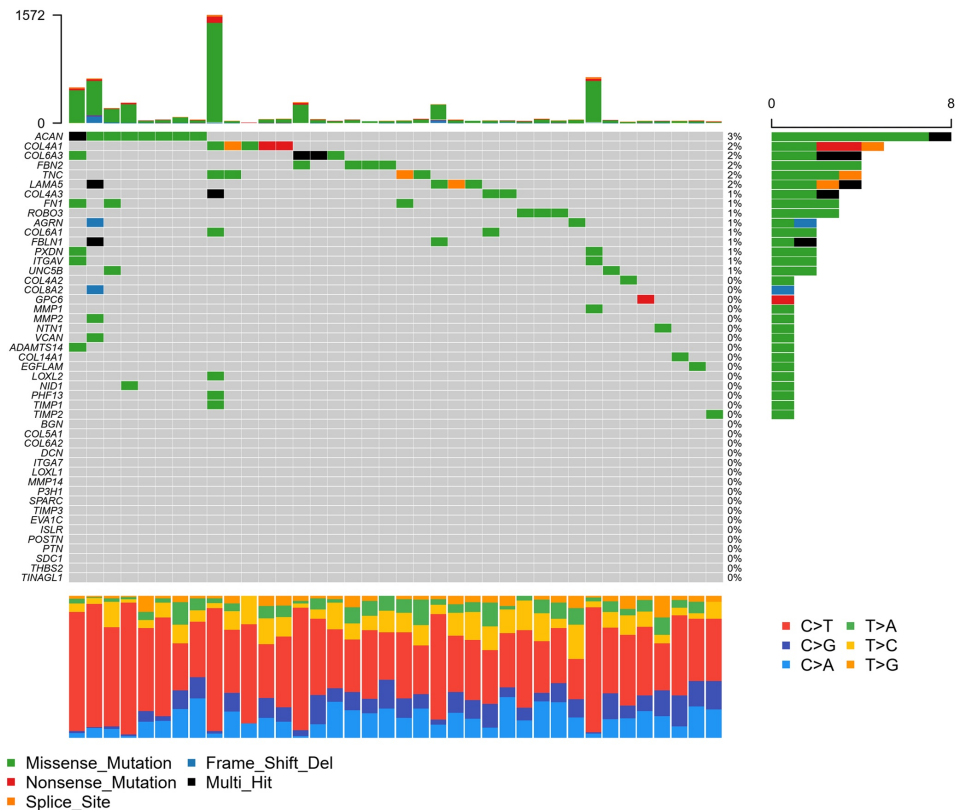

consensus matrix k=2

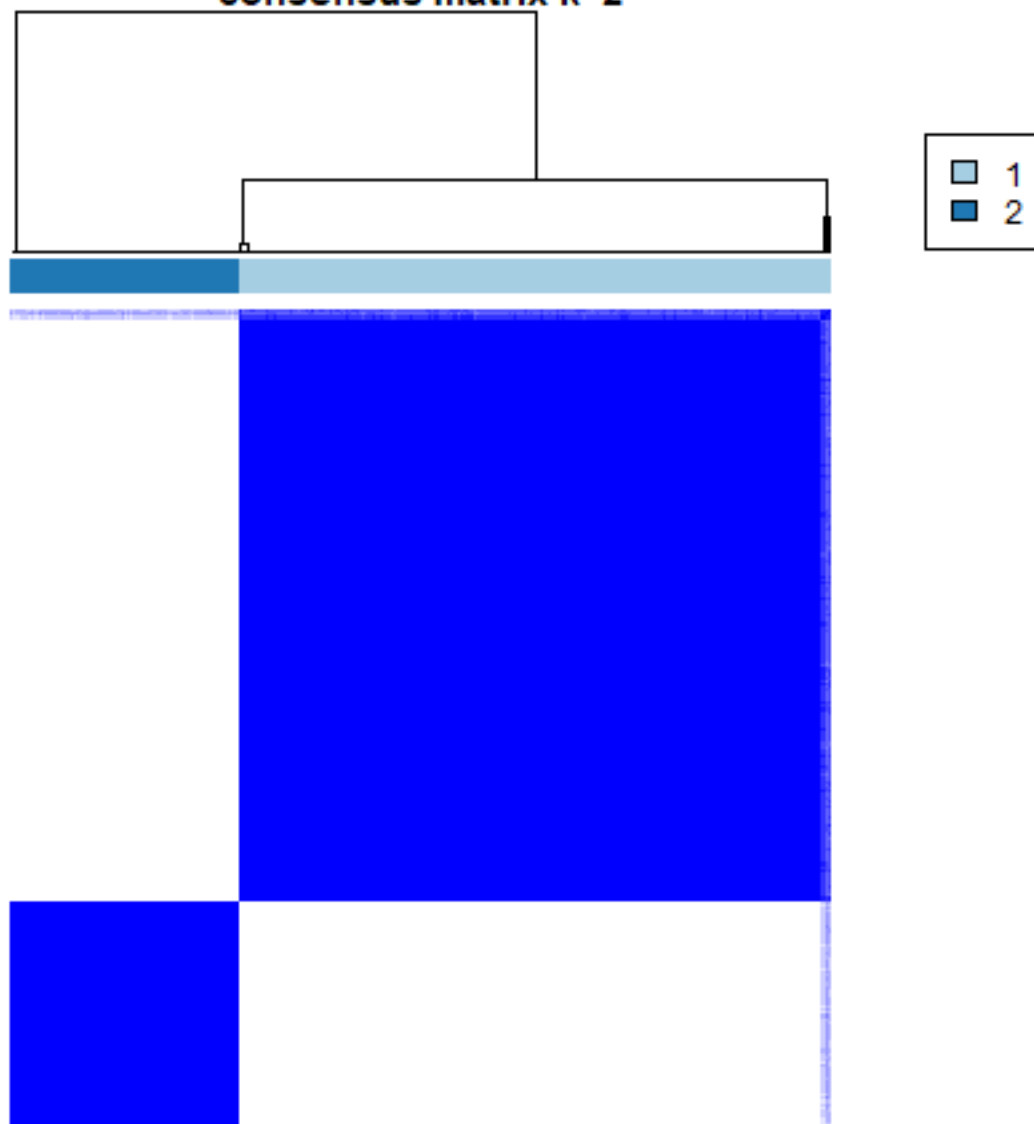

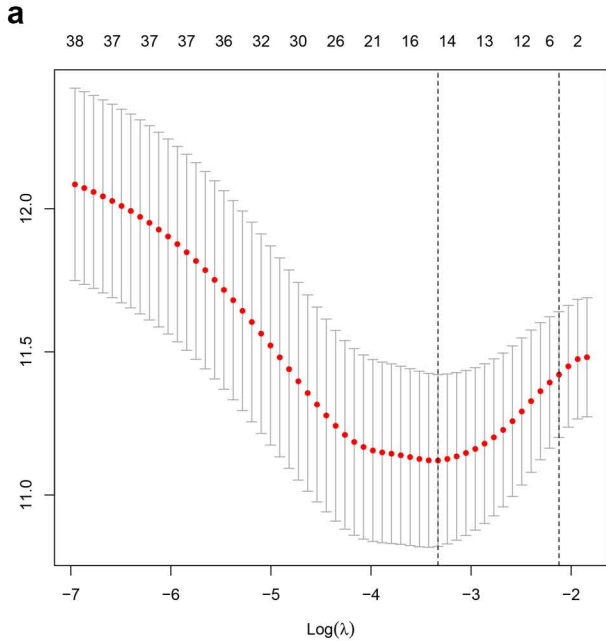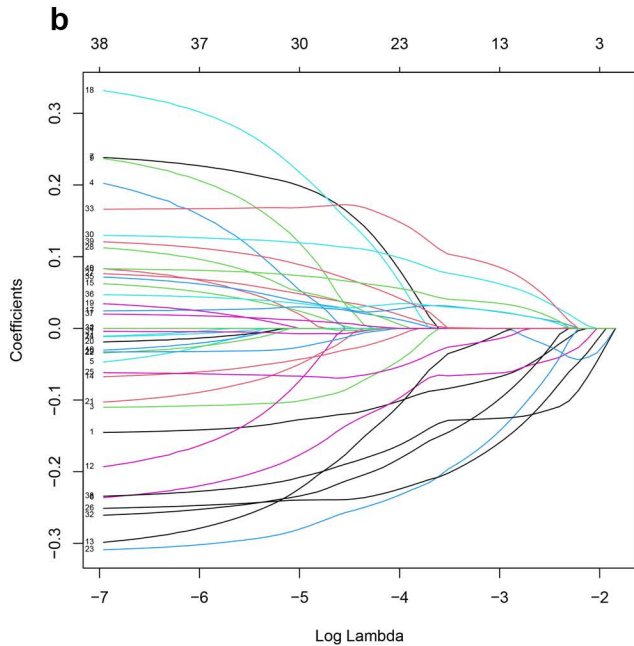

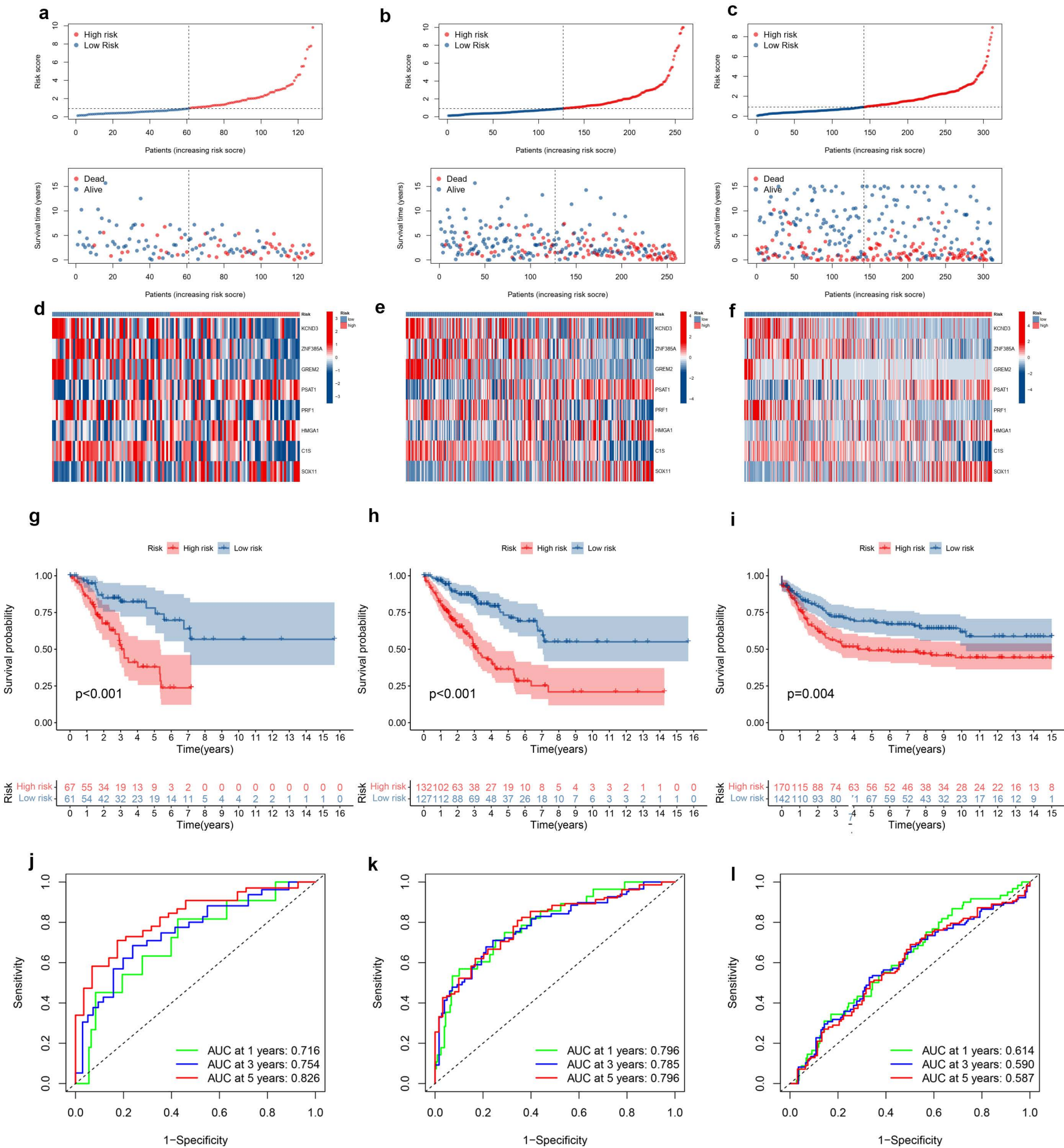

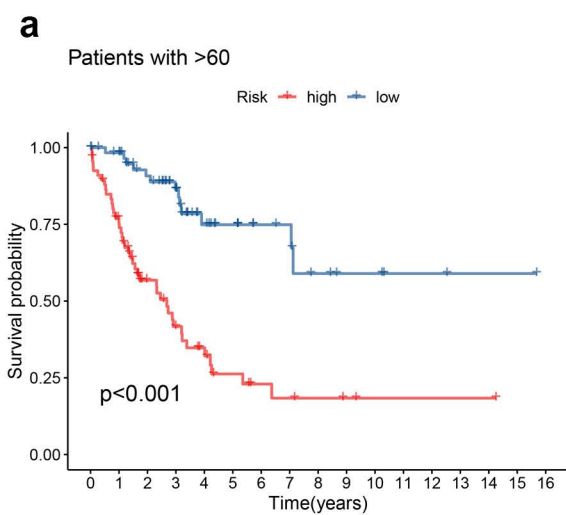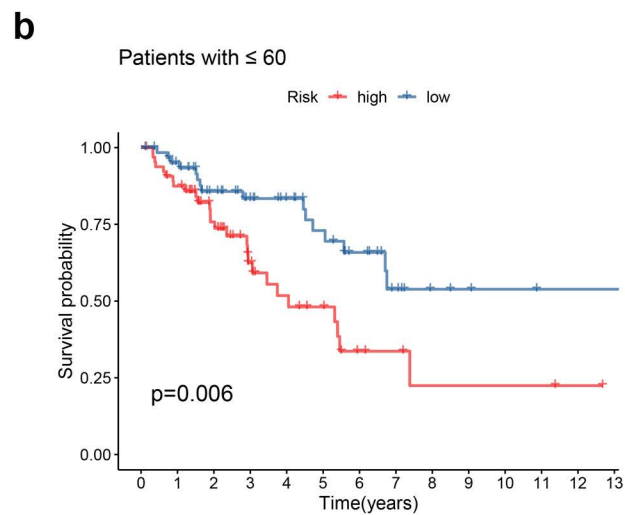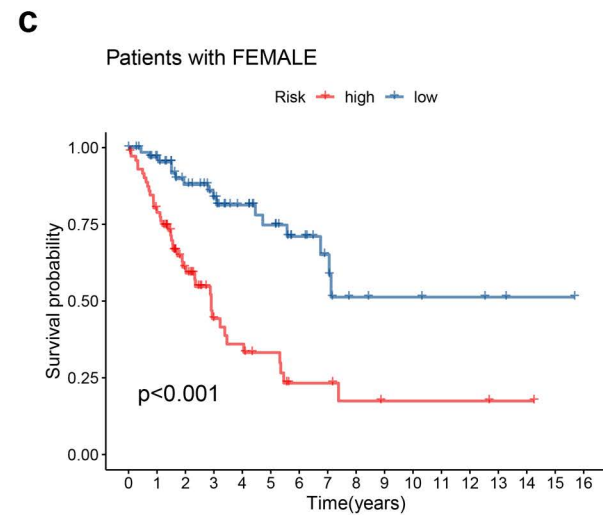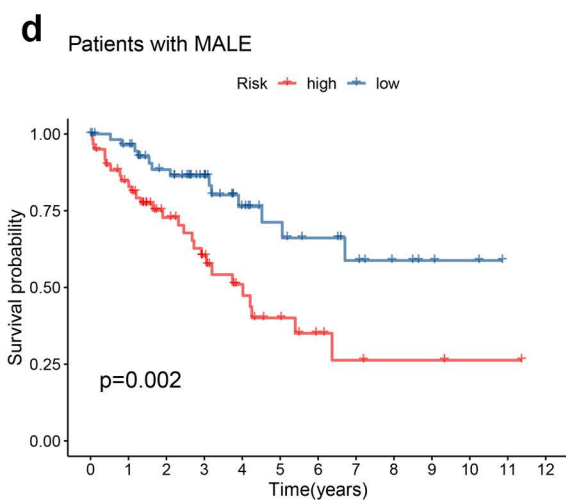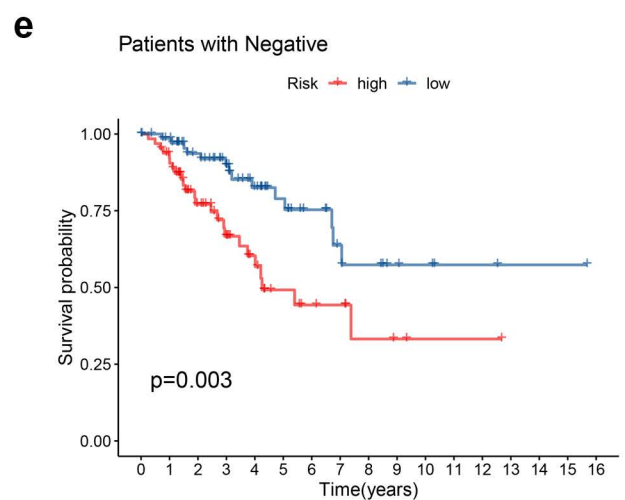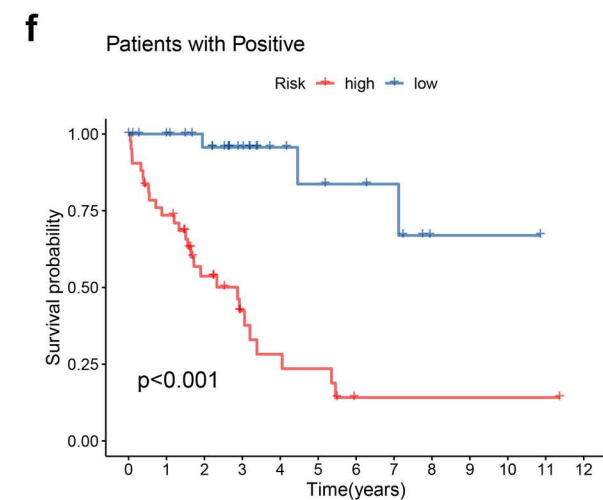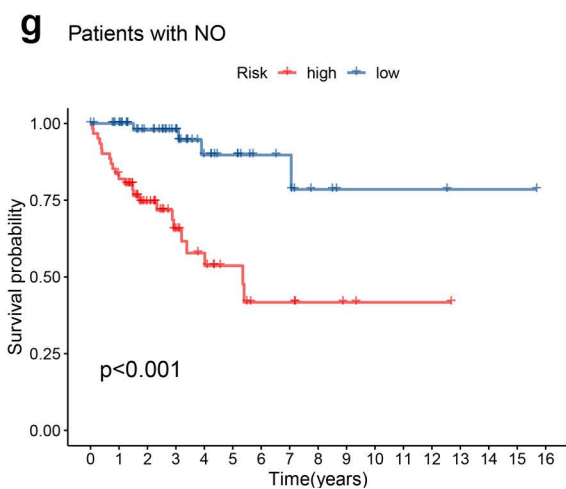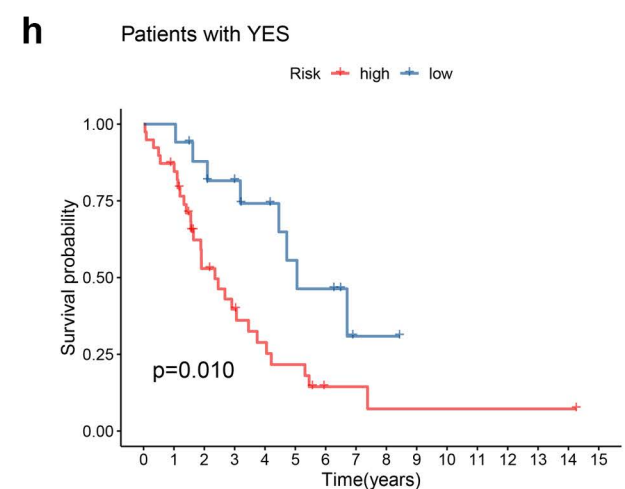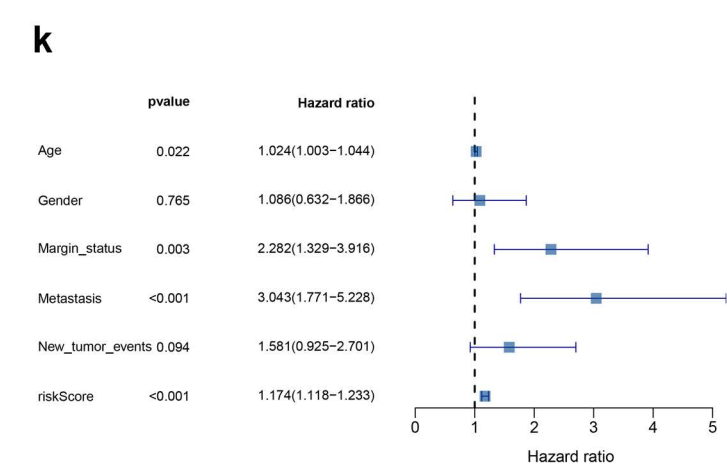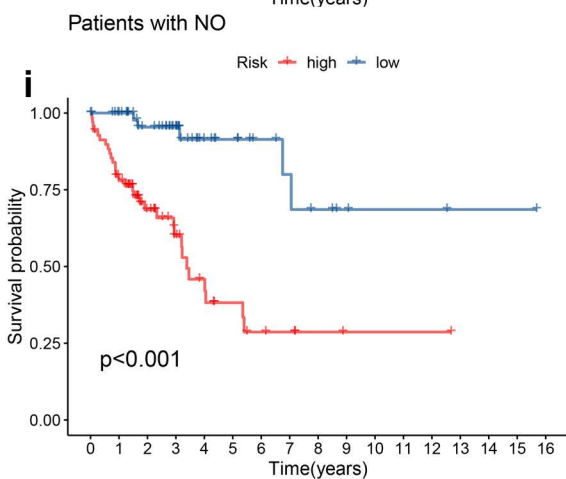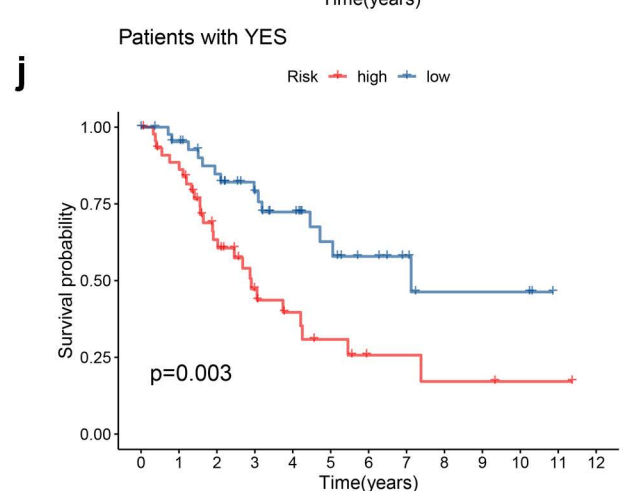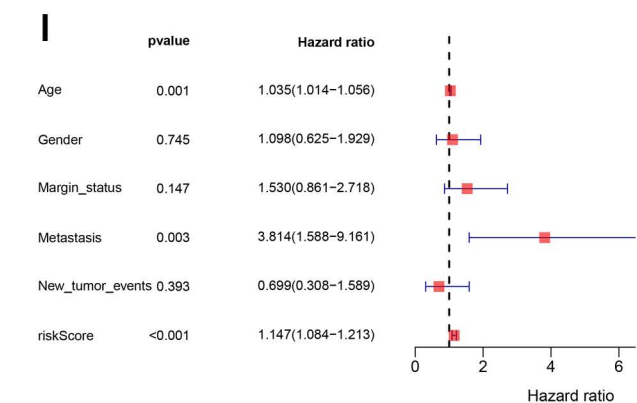

**a**

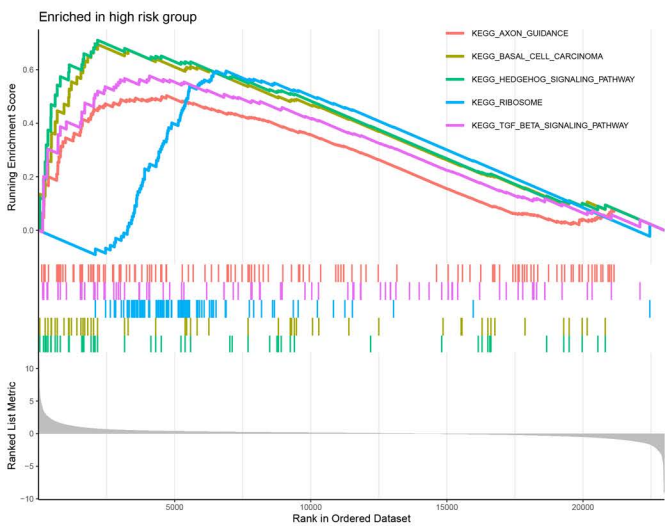

**b**

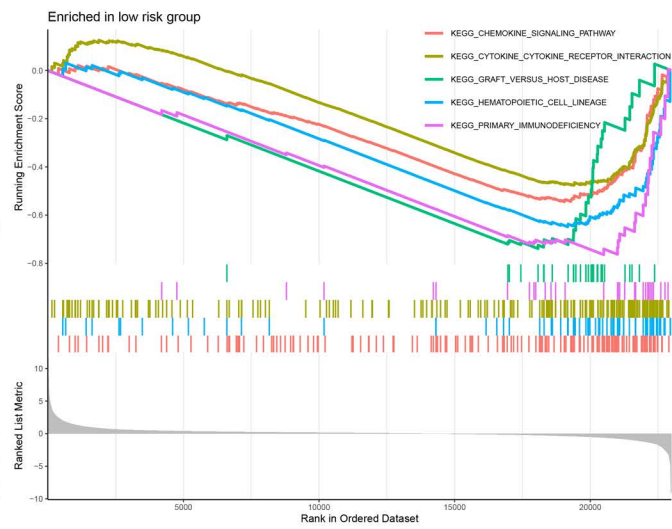

Supplement: Supplementary file 1 — Supporting information. [file IID3-12-e70037-s003.PDF]
