# Supplementary material for: Comprehensive analysis of prognostic and immunological role of basement membrane‐related genes in soft tissue sarcoma
Source: Immun Inflamm Dis. 2024 Oct 11;12(10):e70037. doi: 10.1002/iid3.70037 (PMC11467964; doi:10.1002/iid3.70037)
Supplement: Supplementary file 3 — Supporting information. [file IID3-12-e70037-s001.docx]

**Figure S1.** Mutation frequencies of BMRGs, from the TCGA cohort.

**Figure S2.** Identification of gene subtypes based on DEGs among two subtypes.

**Figure S3.** Identifying representative candidate prognostic genes. (a) The LASSO regression analysis. (b) The Partial likelihood of deviance on the prognostic genes

**Figure S4.** Validation of risk score. (a-c) Ranked dot and scatter plots showing the risk score distribution and patient survival status in testing, entire, and GSE71118 cohorts, respectively. (d-f) Kaplan–Meier analysis of the OS between the two groups in testing, entire, and GSE71118 cohorts, respectively. (g-i) Heatmap of the eight BMRGs expressions in testing, entire, and GSE71118 cohorts, respectively. (j-l) ROC curves to predict the sensitivity and specificity of 1-, 3-, and 5-survival according to the risk score in testing, entire, and GSE71118 cohorts, respectively.

**Figure S5.** Stratification analysis of the risk score in STS. (a-b) Age (age > 60 and age ≤ 60 years old). (c-d) Gender (female and male). (e-f) Margin status (negative and positive). (g-h) Metastasis (no and yes). (i-j) New tumor event (no and yes). (k) Univariate analyses showed the prognostic value of the risk score in the training set. (l) Multivariate showed the prognostic value of the risk score in the training set.

**Figure S6.** The GSEA analysis results between the high-risk group and low-risk group. (a) GSEA of the top 5 pathways significantly enriched in the high-risk group. (b) GSEA of the top 5 pathways significantly enriched in the low-risk group.
